# Confirmatory Factor Analysis of the Inventory of Personality Organization-Reality Testing Subscale

**DOI:** 10.3389/fpsyg.2018.01116

**Published:** 2018-07-05

**Authors:** Neil Dagnall, Andrew Denovan, Andrew Parker, Kenneth Drinkwater, R. Stephen Walsh

**Affiliations:** Department of Psychology, Manchester Metropolitan University, Manchester, United Kingdom

**Keywords:** bifactor model, confirmatory factor analysis, invariance testing, Inventory of Personality Organization, reality testing

## Abstract

The reality testing dimension of the Inventory of Personality Organization, the IPO-RT, has emerged as an important index of proneness to reality testing deficits. However, to date few studies have examined the factorial structure of the IPO-RT in isolation. This is an important and necessary development because studies use the IPO-RT as a discrete measure. Additionally, psychometric evaluation of the IPO suggests alternative factorial solutions. Specifically, recent work supports multidimensionality, whereas initial IPO assessment evinced a unidimensional structure. Accordingly, this study, using a heterogeneous sample (*N* = 652), tested the fit of several factorial models (one-factor, four-factor oblique, second-order, and bifactor) via maximum likelihood with bootstrapping due to multivariate non-normality. Analysis revealed superior fit for the bifactor solution (correlated errors) (CFI = 0.965, SRMR = 0.036, RMSEA = 0.042). This model comprised a general reality testing dimension alongside four subfactors (auditory and visual hallucinations, delusional thinking, social deficits, and confusion). Inter-factor correlations were in the moderate range. Item loadings and omega reliability supported the notion that the IPO-RT emphasizes a single latent construct. The model demonstrated invariance across gender and partial age invariance. Overall, from a psychometric perspective, the IPO-RT functioned effectively at both global and, to an extent, factorial levels. Findings recommend that the IPO-RT should be scored as a total scale, and rather than treat subscales independently, future studies should consider examining factor variance alongside overall scale scores.

## Introduction

### Inventory of Personality Organization (IPO)

Researchers in discrete but related areas (i.e., psychopathology, personality, individual differences and parapsychology) commonly use the Inventory of Personality Organization (IPO) (Lenzenweger et al., 2001) to assess personality functioning (e.g., Prunas and Bernorio, 2016; Espinosa and Rudenstine, 2018). The IPO is a self-report measure that identifies and classifies personality pathology within clinical and non-clinical samples (Smits et al., 2009; Preti et al., 2015). The inclusion of the IPO in published research and translation of the IPO into several different languages (e.g., Dutch, Berghuis et al., 2009; Japanese, Igarashi et al., 2009; Canadian French, Normandin et al., 2002; Brazilian, Oliveira and Bandeira, 2011; Portuguese, Barreto et al., 2017; and Italian, Preti et al., 2015) evidence the measure’s importance.

Conceptually, the IPO derives from Kernberg’s (1984, 1996) psychodynamic model, which has a solid theoretical and diagnostic base. Kernberg (1984, 1996) proposed that general personality disorder originates from a convergence of neurobiologically mediated (e.g., temperament and aggression) and environmentally moderated (e.g., trauma and neglect) factors (Kernberg and Caligor, 2005). Within this conceptualization, personality organization determines pathology. Specifically, Kernberg (1984, 1996) postulated that position on the neurotic, borderline and psychotic realms determined level of personality dysfunction (Smits et al., 2009). Accordingly, the IPO defines level of personality disorder organization via three dimensions: reality testing, predominance of primitive psychological defenses and identity diffusion (Lenzenweger et al., 2012). Personality disorder manifests as a combination of these dimensions plus level of severity (Kernberg, 1996).

Each IPO dimension possesses its own unique attributes and the importance of these to psychological functioning and behavior is well-documented (Kernberg, 1975, 1984). Reality testing denotes, “the capacity to differentiate self from non-self, intrapsychic from external stimuli, and to maintain empathy with ordinary social criteria of reality” (Kernberg, 1996, p. 120). Extreme reality testing failure manifests as psychotic disorganization of thought and behavior. A fuller discussion of reality testing appears later.

Primitive psychological defenses represent protective propensities that distort and interfere with interpersonal interactions (e.g., splitting) (Wolfe and Mash, 2006). Splitting occurs in situations where individuals respond to emotional conflict or stressors by compartmentalizing opposite affect states (Koenigsberg et al., 2001). This process produces ambivalence because it is impossible to integrate contrasting affective states into a cohesive image of self or others. Hence, image perception alternates between polar opposites and the individual excludes balanced views from emotional awareness. Primitive psychological defenses are protective inclinations associated with more severe psychopathology (i.e., projection, denial, dissociation or splitting), which are distinct from healthier defenses (i.e., reaction formation, isolation, undoing, suppression, and repression) (Lenzenweger et al., 2001).

Finally, identity diffusion refers to the failure to develop a distinct identity. Explicitly, lack of differentiated and integrated representations of the self and others (Sollberger et al., 2012). Typically, characteristics, such as lack of cohesion in the subjective experience of self, boundary confusion and fragmentation, are important features of identity diffusion. These manifest as difficulties with internalized value systems (norms, interests, ethics, and ideals) (Sollberger et al., 2012).

Studies assessing the psychometric properties of the IPO using clinical and non-clinical samples usually detail good internal consistency and test–retest reliability (Lenzenweger et al., 2001; Normandin et al., 2002). Illustratively, Foelsch et al. (2000, Unpublished) reported that IPO dimensions displayed satisfactory internal consistency: reality testing, α = 0.85–0.87; primitive defenses, α = 0.80–0.87; and identity diffusion, α = 0.84–0.90. Correspondingly, Lenzenweger et al. (2001) observed comparable coefficient alphas (reality testing: study 1, α = 0.88, study 2, α = 0.87; primitive defenses: study 1, α = 0.81, study 2, α = 0.81; and identity diffusion, study 1, α = 0.88, study 2, α = 0.88). Within study 1, analysis of IPO subscale means across gender failed to reveal significant sex differences.

The IPO demonstrates also temporal stability. Foelsch et al. (2000, Unpublished) reported satisfactory short-term test–retest for subscales in a sample of community adults (reality testing, *r* = 0.80; primitive defenses, *r* = 0.81; and identity diffusion, *r* = 0.83). Lenzenweger et al. (2001) supported this finding. Four-week test–retest correlations were: reality testing, *r* = 0.73; primitive defenses, *r* = 0.72; and identity diffusion, *r* = 0.78. Similarly, factors with the Dutch IPO translation (IPO-NL) demonstrated adequate 1-month test–retest (reality testing, *r* = 0.85; primitive defenses, *r* = 0.82; and identity diffusion, *r* = 0.86) (Berghuis et al., 2009).

Additionally, IPO analysis typically reveals moderate relationships between IPO dimensions (Lenzenweger et al., 2001; Normandin et al., 2002). For instance, factor intercorrelations within Lenzenweger et al.’s (2001) paper were as follows: Primitive Defenses – Identity Diffusion, *r* = 0.82, *p* < 0.001, *r* = 0.83, *p* < 0.001; Primitive Defenses – Reality Testing, *r* = 0.65, *p* < 0.001, *r* = 0.76, *p* < 0.001; and Identity Diffusion – Reality Testing, *r* = 0.62, *p* < 0.001, *r* = 0.73, *p* < 0.001.

Within the three-factor solution, issues arise from the fact that the IPO-RT (the final dimension to emerge) explains insufficient independent variance. Consequently, literature advocates an alternative two-factor solution, where primitive psychological defenses and identity diffusion appear within a clustered dimension and reality testing forms a second factor (Berghuis et al., 2009). Alternatively, Ellison and Levy (2012) recommend a four-factor model, where dimensions represent instability across a range of domains: sense of self and other, goals, behaviors and psychosis. This structure acknowledges that key elements of personality organization, particularly those pertaining to representations of self and others, do not adequately fit a three-factor model (Ellison and Levy, 2012).

Discrepancies in IPO structure may arise from the use of different statistical procedures across studies. In the key psychometric evaluation of the measure, Lenzenweger et al. (2001) confirmed the superior fit of the three-factor solution (vs. alternative two-factor, one-factor, and null models) by conducting a series of confirmatory factor analyses. Whereas, Ellison and Levy (2012) scrutinized factor structure and criterion relations via exploratory structural equation modeling and multiple regression. Item adaptation within translation papers and modifications to item number as part of IPO evolution further complicate structural interpretation. For instance, Berghuis et al. (2009) investigated the dimensionality of the IPO-NL by means of principal component analysis with varimax rotation. In the case of production of shortened/abridged forms of the IPO, Verreault et al. (2013) tested the factorial structure of a 20-item abbreviated version using confirmatory factor analyses. Correspondingly, Smits et al. (2009) used confirmatory factor analyses to develop the IPO-R, a shortened version of the IPO.

### Inventory of Personality Organization-Reality Testing (IPO-RT) Subscale

The current paper examined the content and factorial structure of the reality testing dimension of the IPO (IPO-RT). This was necessary because the IPO-RT has developed into a standalone measure of proneness to reality testing deficits (see Drinkwater et al., 2012; Dagnall et al., 2015). Use of the IPO-RT as a standalone measure dates back to Irwin’s investigation of the relationship between reality testing and belief in the paranormal (Irwin, 2003, 2004). In his formative study, Irwin (2003) employed the Bell Object Relations and Reality Testing Inventory (BORRTI) (Bell et al., 1985; Bell, 1995). Using BORRTI Irwin (2003) reported that paranormal beliefs predicted the tendency to distort internal and external reality. Subsequent consideration of BORRTI revealed that the measure explicitly indexed paranormal content (Irwin, 2003, 2004). This was also true of the other established measure of reality testing, the Borderline Personality Inventory (Leichsenring, 1999). Noting conceptual overlap between BORRTI and the Revised Paranormal Belief Scale (RPBS), Irwin (2003, 2004) cautioned that shared variance might have inflated the relationship between reality testing and belief in the paranormal. Consequently, Irwin (2004) adopted the IPO-RT because it was free of explicit paranormal content.

In the context of belief in the paranormal, several researchers view the IPO-RT as a measure of information processing style rather than psychotic phenomena. This judgment derives from the notion that the IPO-RT provides a representative assessment of evaluative processes as defined by Langdon and Coltheart (2000) explanation of belief generation (Irwin, 2004).

Initial psychometric evaluation of the IPO-RT suggested the subscale was unidimensional. Acknowledging this, Irwin (2004) stated that although the subscale indexes a range of reality testing aspects the IPO-RT probably provides an oversimplification of domain content. Recent findings have challenged the notion that the IPO-RT is unidimensional by identifying potential underlying dimensions (Dagnall et al., 2017). Dagnall et al. (2017), in their study examining the cognitive-perceptual basis of belief in urban legends and the paranormal, performed an exploratory factor analysis with oblique (promax) rotation on the IPO-RT. Exploratory factor analysis advocated a multidimensional four-factor solution accounting for 55% of variance, which was supported via confirmatory factor analysis (CFA). The identified factors were consistent with the theoretical underpinnings of reality testing deficits (Bell et al., 1985; Caligor and Clarkin, 2010). Emergent factors were factor 1, ‘hallucinations’ (auditory and visual); factor 2, ‘delusional thinking’ (beliefs contrary to reality); factor 3, ‘social deficits’ (difficulties reading social cues); and factor 4, sensory/perceptual ‘confusion’ (inability to understand feelings and sensations).

The suggestion that reality testing is multidimensional rather than unitary is not new. For example, Ellison and Levy (2012) using exploratory structural equation modeling found IPO-RT items split into two clusters. Their ‘psychosis’ factor most closely corresponded to the IPO-RT and contained items restricted largely to the pathological pole of reality testing (i.e., hallucinations and delusions) (Kernberg, 1975). Questions related to milder forms of reality testing difficulties, specifically maintaining a grasp on reality testing (e.g., “I can’t tell whether I simply want something to be true, or whether it really is true”), loaded on the ‘instability of self and others’ factor.

### The Present Study

The current paper assessed the psychometric structure of the IPO-RT in isolation. This is important for two main reasons. Firstly, several recent papers have used the IPO-RT as a standalone measure of proneness to reality testing deficits (e.g., Dagnall et al., 2015). Hence, it is important to examine how the IPO-RT functions in this specific context. Secondly, the scale structure from a reality testing perspective will be unaffected by other IPO subscales. Hence, the analysis permitted a cleaner, uncontaminated evaluation of IPO-RT content. Clearly, shared variance within the IPO structure is likely to influence subscale loadings. This was evident within the Ellison and Levy (2012) paper.

Accordingly, an assessment of IPO-RT model fit was undertaken. This compared unidimensional (Lenzenweger et al., 2001) vs. multidimensional structure (Dagnall et al., 2017) via consideration of a progressive hierarchy of competing models. Specifically, a one-factor model for a strict unidimensional assessment, a correlated multidimensional solution testing Dagnall et al. (2017) model, a second-order solution examining whether a latent general reality testing factor existed in addition to multiple dimensions, and a bifactor model examining the unidimensional vs. multidimensional argument in a single analysis (Reise et al., 2010). Bifactor models depict factors as orthogonal. Additionally, bifactor models assess the relative strength of a general underlying factor in comparison to multiple factors (Reise et al., 2010).

Subsequent analysis evaluated IPO-RT structure stability using invariance testing. Explicitly, an assessment of invariance in relation to age and gender. Related studies have tended to focus on the IPO as a composite scale and failed to consider IPO-RT invariance (Verreault et al., 2013). Establishing invariance across groups indicates that observed mean differences are unlikely to be an artifact of measurement bias, and instead reflect true mean differences (Denovan et al., 2017).

Invariance testing is an important means of assessing IPO-RT performance across sub-groups. Consistent with previous research on related measures (e.g., Bell et al., 1985; Preti et al., 2015), invariance tests assessed age and gender. Specifically, Bell et al. (1985) tested for age and gender bias in relation to the Bell Reality Testing Inventory, and Preti et al. (2015) assessed gender invariance for the IPO. Gender, in particular, is an important factor to consider when scrutinizing the psychometric properties of the IPO-RT because gender differences should technically not exist if the measure is an accurate index of personality pathology according to Kernberg’s object-relations model (Kernberg, 1984). In addition to established gender invariance for related measures (Preti et al., 2015), research typically reports non-significant mean gender differences on the IPO-RT (Lenzenweger et al., 2001).

## Materials and Methods

### Participants

Merging independent IPO-RT data sets from previously published studies (Dagnall et al., 2014, 2017) and articles in production created a heterogeneous sample of 652 respondents. Mean (*M*) sample age was 28.63 years (*SD* = 12.41, range = 18–87 years). Sample disaggregation by gender specified that 245 (38%) respondents were male (*M* age = 30.51, *SD* = 13.59, range = 18–87 years) and 407 (62%) were female (*M* age = 27.50, *SD* = 11.52, range = 18–77 years). Recruitment was via emails to staff and students (undergraduate and postgraduate) enrolled on healthcare programs (Nursing, Physiotherapy, Psychology, Speech and Language Therapy, etc.) at a United Kingdom university, and local businesses. Participation occurred between January 2014 and September 2016 (see “Ethics” section). Instructions prevented multiple responses by informing participants not to complete the study if they have participated in similar research.

Several researchers have previously evaluated scale structure using this approach. For instance, Lange et al. (2000) top-down purification of RPBS; Roets and Van Hiel (2011), Need for Closure Scale validation; and Drinkwater et al. (2017), assessment of RPBS dimensionality.

### Materials

The only study measure was the IPO-RT (Lenzenweger et al., 2001). This is the reality testing subscale of IPO (Lenzenweger et al., 2001), which is used frequently as a standalone scale to assess proneness to reality testing deficits (Irwin, 2004; Dagnall et al., 2017). Specifically, the IPO-RT indexes “the capacity to differentiate self from non-self, intrapsychic from external stimuli, and to maintain empathy with ordinary social criteria of reality” (Kernberg, 1996, p. 120). This conceptualization is congruent with Langdon and Coltheart (2000) account of belief generation, which emphases information-processing style rather than psychotic symptomology (Langdon and Coltheart, 2000; Irwin, 2004). The IPO-RT comprises 20-items presented as statements (e.g., “I can’t tell whether certain physical sensations I’m having are real, or whether I am imagining them”). Respondents indicate agreement to each statement via a five-point Likert scale (1 = never true to 5 = always true), hence total IPO-RT scores range from 20 to 100; higher scores indicate proneness to report experiences of reality testing deficits. The IPO-RT possesses construct validity, good internal consistency and test–retest reliability indicating it is a largely psychometrically sound measure (Lenzenweger et al., 2001). However, the fact that studies have failed to establish factor invariance across countries limits generalizability across national samples. Particularly, it suggests cultural differences in interpretations and comprehensions of IPO items (Tucker et al., 2006).

### Procedure

Respondents completed the IPO-RT alongside measures assessing anomalous beliefs, cognitive-perceptual personality factors and decision-making. The basic procedure across studies was standardized. Before taking part, the researchers presented prospective respondents with detailed background information. The brief outlined the nature of the study and delineated ethical procedures. If respondents agreed to participate, they registered informed consent and received the materials. Procedural instructions then directed respondents to consider questions carefully; work through the items systematically, at their own pace; respond to all questions; and answer in an honest and open manner. Questionnaire section order rotated in order to prevent order effects. Alongside item endorsement respondents forwarded basic demographic information (preferred gender, age, etc.).

### Ethics

As preparation for grant bids (September 2012, 2014, and 2016) the researchers obtained ethical authorization for a series of studies investigating anomalous beliefs, cognitive-perceptual personality factors and decision-making. Each submission was “routine” and accordingly ratified (methodological and ethical) by the Director of the Research Institute for Health and Social Change (Faculty of Health, Psychology and Social Care) within Manchester Metropolitan University. This is was the required level of ethical clearance. Additionally, prior to submission, research proposals are peer-reviewed by members of the Professoriate (or equivalent). This process includes ethical and methodological scrutiny. Finally, the Head of the Psychology Department sanctioned the projects. Formal submission to a university ethics panel is not an institutional requirement for routine studies.

### Data Analytic Plan

Prior to specifying and testing competing factor models of the IPO-RT, data screening for outliers and normality occurred. Inter-correlations assessed preliminary relationships among IPO-RT total and subfactor scores. CFA, using AMOS 24, examined the proposed measurement models and determined which best fitted the IPO-RT data. These comprised the unidimensional model advanced by Lenzenweger et al. (2001) and variants of the multidimensional structure identified by Dagnall et al. (2017). The multidimensional model contained four subfactors: ‘auditory and visual hallucinations’ (items 7, 9, 16, 8, 2, 5), ‘delusional thinking’ (items 19, 12, 14, 18, 15, 17, 11), ‘social deficits’ (items 13, 10, 20, 4), and ‘confusion’ (items 1, 3, 6). The potential presence of these factors suggested three alternative models (correlated, second-order, and bifactor).

The correlated four-factor model assumed that reality testing was multidimensional and explained by obliquely related dimensions. Contrastingly, the second-order model derived from the notion that factors were uncorrelated and representative of a general reality testing construct. Finally, the bifactor model reconciled the unidimensional and multidimensional alternatives by advocating that IPO-RT items loaded on four subfactor dimensions and a general factor.

Model parameter appraisal used maximum likelihood (ML) estimation. Multiple indices including chi-square test, Comparative Fit Index (CFI), Standardized Root-Mean-Square Residual (SRMR), and Root-Mean-Square Error of Approximation (RMSEA) evaluated model fit. Using a range of indices ensures robust assessment of model fit.

Generally, non-significant chi-square signifies good data-model fit. However, the statistic is sensitive to sample size and thus insufficient as a standalone CFA index (Byrne, 1994). Accordingly, model evaluation referenced also CFI, SRMR, and RMSEA. Good fit thresholds for these indices are CFI > 0.90, SRMR < 0.08 and RMSEA < 0.08 (Browne and Cudeck, 1993). A CFI above 0.87 and SRMR and RMSEA values below 0.10 indicate marginal fit (Bong et al., 2013). For RMSEA the 90% confidence interval (CI) was included. Additionally, for model comparison analysis considered Akaike’s Information Criterion (AIC), with lower values indicative of superior fit.

For each model, Modification Indices (MI) indicated the extent chi-square would improve if constrained parameters covaried. MI values higher than 20 related to subfactor items were inspected (Rossier et al., 2012). Although, statisticians typically recommend against covarying subfactor item errors, covariance in the present study was justified because some subfactor items possessed similarities in item content (Byrne, 2010).

Following model specification and testing, Cronbach’s alpha examined internal consistency of the IPO-RT. In addition to alpha, coefficient omega (*ω*) and omega hierarchical (*ωh*) considered reliability (estimated with the Omega program; Watkins, 2013), which can more accurately capture the reliability of bifactor solutions (Brunner et al., 2012). Coefficient omega calculates the reliability of a latent factor combining specific and general factor variance. Omega hierarchical calculates the reliability of a latent factor without factoring in the variance from other specific and general factors.

To assess invariance of the superior factor solution, multi-group CFA examined an increasingly restrictive set of models in relation to gender (male vs. female) and age (below 24 vs. above 24). A median split analysis informed the decision to separate the sample at 24 years of age, a method utilized in previous research (Allan et al., 2015). Analysis tested configural, metric and scalar invariance models. Configural invariance assesses the degree to which the same factor structure holds across the groups of interest. Metric invariance examines whether the factor structure and factor loadings are invariant across groups. Scalar invariance examines factor structure, factor loadings and item intercepts. If a measure possesses invariance at the scalar level, mean differences are valid across tested groups and are not a result of measurement bias. When testing invariance in addition to demonstrating satisfactory model fit, CFI values should not change by more than 0.02 (Cheung and Rensvold, 2002). In large samples, due to its sensitivity, use of chi-square as an index for invariance is not advisable (Brown, 2006). Following invariance tests, MANOVA examined mean comparisons among the groups utilized for invariance testing: gender (men vs. women) and age (below 24 years vs. above 24 years).

## Results

### Preliminary Analyses

Data screening prior to analysis identified 16 extreme scores. Excluding these scores left a total sample of 652. The average IPO-RT score was 36.79 (*SD* = 11.58). Kurtosis and skewness scores for the subfactors and total IPO-RT all fell within −2 and +2 (Byrne, 2010) (**Table 1**). An assessment of multivariate normality revealed Mardia (1970) kurtosis coefficient to be 147.212 with a critical ratio of 63.357. The magnitude of this indicates that the data was multivariate non-normal and can result in standard error biases (Bentler and Wu, 2005). Accordingly, analysis used ML estimation with bootstrapping (600 resamples) to generate accurate estimations of standard errors with accompanying confidence intervals (bias-corrected at the 95% confidence level) and *p*-values (Byrne, 2010). Research demonstrates that naïve bootstrapping is a robust alternative to other ML robust methods (e.g., the Satorra–Bentler chi-square), and performs effectively even under conditions of extreme non-normality (Nevitt and Hancock, 2001). The Bollen–Stine bootstrap *p* assessed fit in addition to indices of χ^2^, CFI, SRMR, and RMSEA. Bollen–Stine gauges fit without normal theory limitations (Bollen and Stine, 1992), and *p* > 0.05 suggests excellent global fit.

**Table 1:** Descriptive statistics and intercorrelations for IPO-RT total and subscales.

Variable	*Mean*	*SD*	Skew	Kurtosis	1	2	3	4	5
1. IPO-RT total	36.79	11.58	0.84	0.41		0.86^∗∗^	0.89^∗∗^	0.73^∗∗^	0.74^∗∗^
2. Auditory and visual hallucinations	10.44	4.09	1.15	1.07			0.67^∗∗^	0.48^∗∗^	0.52^∗∗^
3. Delusional thinking	12.17	4.54	0.97	0.49				0.56^∗∗^	0.54^∗∗^
4. Social deficits	6.92	2.76	1.02	0.84					0.44^∗∗^
5. Confusion	7.25	2.73	0.44	−0.36					

*^∗∗^p < 0.001.*

Inter-correlations among all scale items were significant (**Table 2**), and the four subscales identified by Dagnall et al. (2017) possessed moderate to strong relationships (**Table 1**). All inter-correlations were below 0.90 suggesting no multicollinearity (Tabachnick and Fidell, 2001).

**Table 2:** Descriptive statistics and intercorrelations for IPO-RT items.

Item	*Mean*	*SD*	1	2	3	4	5	6	7	8	9	10	11	12	13	14	15	16	17	18	19	20
1	2.82	1.11		0.35	0.53	0.22	0.33	0.39	0.21	0.20	0.23	0.14	0.30	0.22	0.11^∗^	0.20	0.21	0.14	0.19	0.27	0.26	0.19
2	1.92	1.09			0.42	0.39	0.52	0.41	0.48	0.46	0.47	0.29	0.46	0.31	0.23	0.28	0.32	0.38	0.36	0.37	0.24	0.23
3	2.26	1.13				0.37	0.42	0.50	0.30	0.33	0.33	0.31	0.36	0.26	0.24	0.32	0.32	0.23	0.29	0.40	0.28	0.33
4	1.38	0.79					0.30	0.30	0.34	0.34	0.36	0.33	0.35	0.29	0.30	0.29	0.30	0.31	0.34	0.40	0.24	0.33
5	2.38	1.03						0.41	0.40	0.40	0.44	0.34	0.38	0.27	0.28	0.31	0.28	0.29	0.39	0.36	0.22	0.24
6	2.16	1.14							0.33	0.31	0.30	0.32	0.42	0.29	0.26	0.30	0.26	0.23	0.27	0.41	0.27	0.37
7	1.45	0.77								0.67	0.54	0.28	0.35	0.31	0.24	0.32	0.40	0.45	0.38	0.32	0.27	0.22
8	1.41	0.76									0.56	0.27	0.41	0.29	0.26	0.29	0.38	0.43	0.38	0.36	0.27	0.23
9	1.82	1.01										0.29	0.42	0.29	0.24	0.30	0.41	0.53	0.50	0.39	0.30	0.24
10	1.81	0.96											0.38	0.21	0.58	0.39	0.33	0.22	0.28	0.33	0.20	0.44
11	1.65	0.94												0.27	0.29	0.33	0.36	0.40	0.37	0.47	0.31	0.35
12	1.65	0.96													0.17	0.33	0.36	0.24	0.25	0.35	0.58	0.19
13	1.67	0.87														0.31	0.25	0.17	0.23	0.29	0.14	0.44
14	1.68	0.87															0.46	0.27	0.28	0.37	0.34	0.29
15	1.75	1.04																0.39	0.37	0.36	0.40	0.31
16	1.44	0.82																	0.39	0.30	0.24	0.21
17	2.16	1.09																		0.43	0.28	0.30
18	1.71	0.94																			0.39	0.34
19	1.55	0.85																				0.17
20	2.04	1.05																				

*All intercorrelations are significant at p < 0.001, but one which is significant at p < 0.05 (indicated with ^∗^).*

### Confirmatory Factor Analyses

The unidimensional, one-factor model indicated poor fit across all indices but SRMR, χ^2^ (170, *N* = 652) = 1219.653, *p* < 0.001, CFI = 0.780, SRMR = 0.070, RMSEA = 0.100 (CI of 0.092 to 0.103). Bollen–Stine *p* = 0.002, suggesting poor fit. High error covariance existed in more than 50% of the items. Consequently, analysis did not permit item error covariance (Byrne, 2010). The correlated four-factor model demonstrated good fit on all indices but CFI, which indicated marginal fit, χ^2^ (164, *N* = 652) = 735.665, *p* < 0.001, CFI = 0.880, SRMR = 0.062, RMSEA = 0.073 (CI of 0.068 to 0.079). Bollen–Stine *p* = 0.002, suggesting poor fit. However, this could be a function of the large sample used (Cooper, 2017), and analysis considered the Standardized Residual Covariance Matrix. If the estimated model represents good fit, the majority of residual covariances will be less than two (Joreskog, 1993), which was apparent from the results. The four-factor solution possessed incidences of high MI related to items 7 and 8, 2 and 5, 14 and 15, 13 and 10, 19 and 12. Covarying within-item error among these items significantly improved model fit, χ^2^ difference (5, *N* = 652) = 291.614, *p* < 0.001, resulting in good data-model fit (**Table 3**). Although Bollen–Stine *p* = 0.002, the majority of residual covariances were below two. Consultation of factor loadings revealed that items were positive, possessed moderate to high loadings (i.e., above 0.4), and were significant (*p* < 0.05) with lower 95% Confidence Intervals all above 0.5, suggesting all items loaded meaningfully (Arifin and Yusoff, 2016).

**Table 3:** Fit indices for IPO-RT factor models.

Model	χ^2^	*df*	Bollen–Stine *p*	CFI	SRMR	RMSEA (90% CI)	AIC
One-factor	1219.65^∗∗^	170	0.002	0.779	0.070	0.097 (0.092–0.103)	1339.653
Four-factor oblique	735.665^∗∗^	164	0.002	0.880	0.062	0.073 (0.068–0.079)	867.665
Four-factor oblique (CE)	444.050^∗∗^	159	0.002	0.940	0.048	0.052 (0.047–0.058)	586.050
Second-order	739.361^∗∗^	166	0.002	0.880	0.062	0.073 (0.068–0.078)	867.361
Second-order (CE)	473.934^∗∗^	162	0.002	0.934	0.048	0.054 (0.049–0.060)	609.934
Bifactor	390.864^∗∗^	150	0.002	0.949	0.039	0.050 (0.044–0.056)	550.864
Bifactor (CE)	312.125^∗∗^	147	0.003	0.965	0.036	0.042 (0.035–0.048)	478.125

*CE, correlated errors; χ^2^, chi-square goodness-of-fit statistic; df, degrees of freedom; CFI, Comparative Fit Index; SRMR, Standardized Root-Mean-Square Residual; RMSEA, Root-Mean-Square Error of Approximation; AIC, Akaike Information Criterion; ^∗∗^χ^2^ significant at p < 0.001.*

Fit indices for the second-order model suggested good fit on all indices but CFI, which reported marginal fit, χ^2^ (166, *N* = 652) = 739.361, *p* < 0.001, CFI = 0.880, SRMR = 0.062, RMSEA = 0.073 (CI of 0.068 to 0.078). Bollen–Stine *p* = 0.002, suggesting poor fit. In addition, certain items (item 11 and item 4) possessed a majority of residual covariances above two. Similar to the four-factor model, high within-item errors were present for items 7 and 8, 14 and 15, 13 and 10, 19 and 12. Allowing errors to covary significantly improved model fit, χ^2^ difference (4, *N* = 652) = 265.428, *p* < 0.001, resulting in good fit overall. Bollen–Stine *p* = 0.002, suggesting poor fit; however, covarying errors resulted in a noticeably lower incidence of residual covariances above two. Consistent with the four-factor model, factor loadings were moderate to high, positive, and significant (*p* < 0.05). All lower 95% Confidence Intervals were greater than 0.5.

**FIGURE 1:**
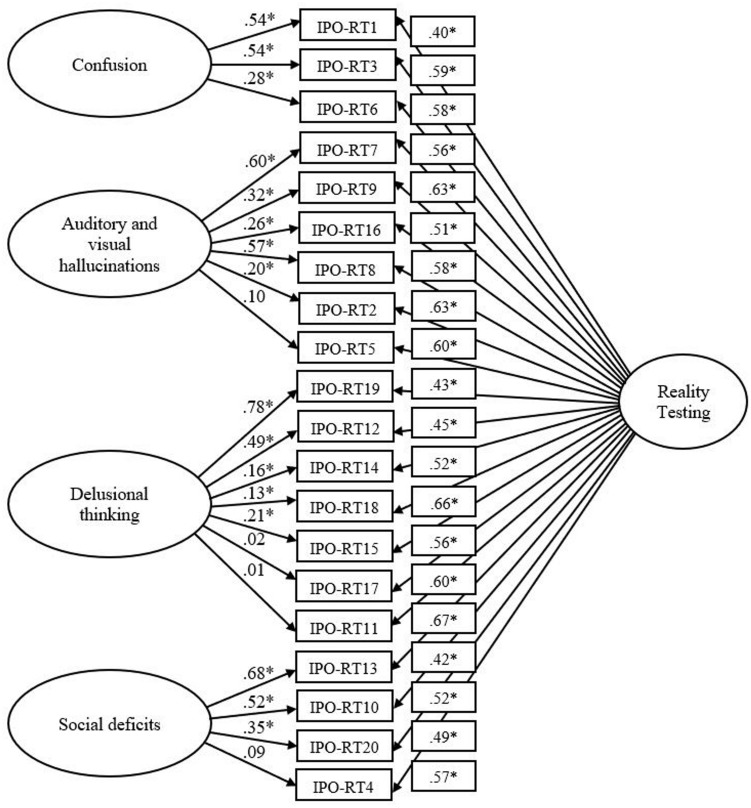
Bifactor IPO-RT model. Latent variables are represented by ellipses; measured variables are represented by rectangles; error is not shown, but was specified for all variables. Error covariances between IPO-RT9 and IPO-RT16, IPO-RT2 and IPO-RT5, IPO-RT14 and IPO-RT15 are not shown but were included in the analysis. ^∗^*p* < 0.05 (using bootstrap significance estimates).

The bifactor solution possessed good data-model fit across all indices, χ^2^ (150, *N* = 652) = 390.864, *p* < 0.001, CFI = 0.949, SRMR = 0.039, RMSEA = 0.050 (CI of 0.044 to 0.056). Bollen–Stine *p* = 0.002, suggesting poor fit; however, the majority of residual covariances exceeded two. For this model, items 9 and 16, 2 and 5, 15 and 14 possessed high within-item error. Correlating error terms resulted in a significant improvement in overall fit, χ^2^ difference (3, *N* = 652) = 78.740, *p* < 0.001). Bollen–Stine *p* = 0.003, suggesting poor fit; however, the majority of residual covariances exceeded two. A comparison of AIC statistics among the tested models revealed that the bifactor solution with correlated errors demonstrated superior fit (**Table 3**). The parameter estimates for the bifactor model demonstrated moderate to high factor loadings (i.e., >0.4) for all items relative to either a general factor or a subfactor (**Figure 1**). Specifically, loadings on the general factor were all greater than 0.4 and significant (*p* < 0.05), with lower 95% Confidence Intervals greater than 0.5. However, loadings on the subfactors did not all meet this threshold, specifically items 17 (*p* = 0.643 [−0.082 to 0.114]), 11 (*p* = 0.801 [−0.102 to 0.108]), 5 (*p* = 0.062 [−0.003 to 0.203]), and 4 (*p* = 0.154 [−0.032 to 0.219]). This suggests that these items more directly predict general reality testing rather than delusional thinking.

### Assessment of Reliability

Internal consistency for IPO-RT total was excellent (α = 0.904). Internal reliability was also good for auditory and visual hallucinations (α = 0.832), delusional thinking (α = 0.800), and was satisfactory for social deficits (α = 0.729) and confusion (α = 0.726). Coefficient omega reported consistent results to alpha reliability: excellent reliability for a general factor (IPO-RT) (*ω* = 0.929), satisfactory to good reliability for auditory and visual hallucinations (*ω* = 0.844), delusional thinking (*ω* = 0.827), social deficits (*ω* = 0.820), and confusion (*ω* = 0.743). Omega hierarchical was similarly high for a general reality testing factor (*ωh* = 0.834); however, suggested lower estimates for auditory and visual hallucinations (*ωh* = 0.214), delusional thinking (*ωh* = 0.146), social deficits (*ωh* = 0.429), and confusion (*ωh* = 0.318). Common variance revealed a similar pattern; IPO-RT total accounted for 62.7% whereas auditory and visual hallucinations, delusional thinking, social deficits, and confusion explained 9.2%, 9.5%, 11.8%, and 6.8%, respectively. The percentage of uncontaminated correlations (PUC) was 76.3%, suggesting a reasonable quantity of correlations reflect general factor variance.

### Measurement Invariance Across Gender and Age

For the bifactor model, assessment of invariance relating to gender and age was undertaken. For gender (male vs. female), a test of configural invariance indicated good data-model fit, χ^2^ (297, *N* = 652) = 534.862, *p* < 0.001, CFI = 0.951, SRMR = 0.077, RMSEA = 0.035 (CI of 0.030 to 0.040). A metric invariance test additionally found good fit, χ^2^ (332, *N* = 652) = 574.778, *p* < 0.001, *p* < 0.001, CFI = 0.950, SRMR = 0.054, RMSEA = 0.034 (CI of 0.029 to 0.038). The difference in CFI between configural and metric models was less than 0.02, providing support for invariance in relation to factor structure and factor loadings. The scalar invariance test reported good fit overall, χ^2^ (352, *N* = 652) = 628.724, *p* < 0.001, CFI = 0.943, SRMR = 0.055, RMSEA = 0.035 (CI of 0.030 to 0.039). The difference in CFI between metric and scalar models was below the threshold of 0.02, indicating the presence of strong factorial invariance. Bollen–Stine, however, inferred poor fit for configural (*p* = 0.008), metric (*p* = 0.015), and scalar (*p* = 0.007) models. An inspection of the Standardized Residual Covariance Matrix revealed the majority of residual covariances were above two, supporting good fit between the model and data.

For the two age groups (below 24 years vs. above 24 years), the configural invariance model suggested good fit, χ^2^ (297, *N* = 652) = 559.910, Bollen–Stine *p* = 0.003, CFI = 0.944, SRMR = 0.047, RMSEA = 0.037 (CI of 0.032 to 0.042). The test for metric invariance also reported good data-model fit, χ^2^ (332, *N* = 652) = 639.708, Bollen–Stine *p* = 0.002, CFI = 0.934, SRMR = 0.057, RMSEA = 0.038 (CI of 0.033 to 0.042). The CFI difference between the test for factor structure and factor loadings was less than 0.02. Results for scalar invariance indicated good fit, χ^2^ (352, *N* = 652) = 767.038, Bollen–Stine *p* = 0.002, CFI = 0.911, SRMR = 0.060, RMSEA = 0.043 (CI of 0.038 to 0.047). However, the CFI difference between scalar and metric models exceeded 0.02, signifying non-invariant intercepts across the two age groups.

Accordingly, a test for partial scalar invariance was undertaken. This examined intercepts on an item-by-item basis and then excluded items with differing intercepts from the invariance testing. This process resulted in identification of the intercepts responsible for the non-invariance. Excluding the constraint for item 2 resulted in a scalar model with a CFI difference less than 0.02 relative to the metric model (0.917 vs. 0.934), supporting partial invariance at the intercept level across the two age groups. Bollen–Stine, however, inferred poor fit for configural (*p* = 0.003), metric (*p* = 0.002), and partial scalar (*p* = 0.002) models. The majority of residual covariances were above two, supporting good fit between the model and data.

### Mean Differences in Reality Testing Across Gender and Age

A MANOVA with bootstrapping (600 resamples) compared IPO-RT total and subfactor scores across gender (245 men; 407 women) and age group (350 below 24 years; 302 above 24 years). The bias-corrected method was used to adjust parameter estimates, standard errors, and effect sizes. Results indicated a significant difference between genders, *Pillai’s trace* = 0.016, *F*(4,645) = 2.680, *p* = 0.031, $\eta_{\text{p}}^{2}$ = 0.016, and between age groups, *Pillai’s trace* = 0.064, *F*(4,645) = 11.066, *p* < 0.001, $\eta_{\text{p}}^{2}$ = 0.064. Univariate ANOVAs revealed men and women differed on the confusion subfactor, *F*(1,648) = 38.880, *p* = 0.018, $\eta_{\text{p}}^{2}$ = 0.009. Bootstrap estimates, using ‘female’ as a reference category, did not support this result, BCa 95% CI of −0.816 to 0.238, *p* = 0.265.

For age, participants differed in relation to IPO-RT total and all subfactors, with the under 24 age group consistently scoring higher than the over 24 age group. Bootstrap estimates, using ‘above 24 years’ as a reference category, supported these differences. For IPO-RT total, BCa 95% CI of 3.612 to 8.270, *p* = 0.002; auditory and visual hallucinations, BCa 95% CI of 1.396 to 2.896, *p* = 0.002; delusional thinking, BCa 95% CI of 0.608 to 2.549, *p* = 0.003; social deficits, BCa 95% CI of 0.075 to 1.138, *p* = 0.022; and confusion, BCa 95% CI of 0.928 to 2.038, *p* = 0.002.

### Analysis Summary

The CFA analyses indicate that the bifactor solution (with correlated errors) explains the data best in terms of model fit statistics. Moreover, the results support invariance for this model across gender and partial invariance across age. The correlated four-factor model and second-order model proved almost as good as one another in relation to data-model fit. The one-factor model did not explain the data well, clearly suggesting that a general reality testing factor on its own does not sufficiently represent these data.

## Discussion

Evaluation of IPO-RT models (one-factor, four-factor, second-order, and bifactor) found superior fit for the bifactor solution (with correlated errors). This model comprised a single general dimension alongside four distinct subfactors (auditory and visual hallucinations, delusional thinking, social deficits and confusion) (Dagnall et al., 2017). Item loadings for the bifactor solution were acceptable at general and to an extent subfactor levels. Alpha and omega coefficients suggested satisfactory to excellent reliability for the general and specific factors. However, omega hierarchical supported the superiority of a general reality testing factor. Examination of subfactor content revealed conceptual coherence; items possessed commonality and related clearly to factor labels. Correlations between subfactors were in the moderate range, the strongest association (*r* = 0.67) was between auditory and visual hallucinations and delusional thinking. These factors represent responses at the pathological pole of the reality testing dimension (Kernberg, 1975). In addition, multi-group CFA suggested that despite the existence of mean differences in reality testing across gender (confusion subfactor only) and age, the superior bifactor model was invariant across gender in terms of factor structure, factor loadings and item intercepts. For age, results supported partial invariance. This indicates that differences in IPO-RT scores are (with the exception of item two pertaining to age) likely to reflect true mean differences as opposed to bias in measurement. Furthermore, gender mean differences were not apparent for IPO-RT total and the majority of subfactors, and the difference for confusion did not exist following bootstrapping. This is consistent with Kernberg’s object-relations model, in which personality pathology indicates no gender differences (Kernberg, 1984).

Adoption of a bifactor IPO-RT model resolves previously reported structural differences and reconciles dimensionality debates (unidimensional vs. multiple factors). Indeed, the range of solutions identified in preceding articles provides support for the bifactor structure. In situations where data index both unidimensional (single common factor) and multidimensional latent (similar domain content) structures, psychometric analysis often produces ambiguity and structural variations (Reise et al., 2010).

With reference to the IPO-RT, this explains why researchers report different factorial solutions. Specifically, Lenzenweger et al. (2001) delineated the IPO-RT as unidimensional, whereas Ellison and Levy (2012) observed that IPO-RT items split between two factors corresponding to severity of reality testing deficit. Explicitly, milder reality testing difficulties loaded on a general ‘instability of self and others’ dimension, whereas items related to psychopathology converged into a separate ‘psychosis’ dimension. Furthermore, when analyzed as a standalone measure, Dagnall et al. (2017) observed the four-factor solution assessed in the current paper. Research with other measures of reality testing has also identified subfactors. For example, Bell et al. (1985) performed a factor analysis on the Bell Reality Testing Inventory and identified three dimensions of reality testing ego function (reality distortion, uncertainty of perception, and hallucinations and delusions).

The proposal of alternative models reflects the fact that measurement of complex psychological/pathological constructs necessitates the inclusion of a broad range of items (Reise et al., 2010). This requirement creates the conceptual paradox where items concurrently assess both a general factor and separate subfactors. In such circumstances second-order and bifactor models best explain data (Chen et al., 2006). This is certainly true of the IPO-RT when researchers use the measure as a standalone index of proneness to reality testing deficits. With reference to the IPO, different solutions may emerge due to shared variance between subfactors and the reality testing construct breadth. This issue of shared variance existed in the present study for the IPO-RT, evident in the sense specific items (i.e., 17, 11, 5, and 4) loaded generally well on their designated factors across solutions but loaded poorly on these factors once they were examined in a bifactor context. A bifactor analysis helped to disentangle whether general vs. specific factors best explained items, revealing that a general factor accounted for the majority of variance. In practice, therefore, the use of unidimensional subscales is not recommended and the validity of such scales is debatable given the majority of variance shared between items pertaining to subscales is attributable to an underlying general factor.

Contrastingly, a one-factor model did not represent these data well, indicating that a general IPO-RT factor is not sufficient to account for all the variance in the measure. In addition, although this study highlights the significance of a general reality testing factor, the proposed subscales by Dagnall et al. (2017) are not completely invalid; a general factor explained the majority of variance, yet the four subfactors accounted for a non-redundant degree of variance. Correspondingly, though the validity of the subscales in isolation is questionable, they could be utilized in combination with total scores when administering the measure. This suggestion is in line with other studies observing greater data-model fit of bifactor solutions that emphasize the importance of a general factor relative to subfactors (e.g., Denovan et al., 2017; McElroy et al., 2018).

In addition, although bifactor modeling is increasingly used in psychological/social sciences and provides an intuitive method of assessing unidimensionality vs. multidimensionality, it has received criticism. Specifically, bifactor models, by virtue of incorporating a general factor that loads onto all items and more free parameters (Murray and Johnson, 2013), can be subject to bias in favor of data-model fit and explained variance compared with traditional CFA solutions. Brouwer et al. (2013) revealed that an element of bias exists (i.e., cross-loadings favored a general factor vs. subfactors), but this effect was marginal. Nonetheless, further research into bias linked with bifactor modeling is apposite (McElroy et al., 2018).

Theoretically, however, a bifactor IPO-RT model is advantageous because it allows researchers to investigate the degree to which general and specific factors predict external variables. This is important since relationships with potentially related factors, such as schizotypy, may vary as a function of the degree to which items index pathology. Indeed, Bell et al. (1985) found only low correlations between reality distortion and uncertainty of perception subscales and most Brief Psychiatric Rating Scale (BPRS) symptom scales. Additionally, schizophrenics, schizoaffectives, and borderlines scored higher on the reality distortion and hallucinations and delusions dimensions. In this context, a bifactor model provides a framework for explaining the degree to which both item commonality and heterogeneity contribute to specific constructs (Gustafsson and Aberg-Bengtsson, 2010). Accordingly, the identification of distinct factors within the reality testing dimension facilitates the development and testing of more sophisticated models.

Generally, the present study demonstrated that the IPO-RT is a psychometrically robust scale that functions as a concise measure of propensity to report reality testing deficits. This provides further validation for studies utilizing the measure previously and subsequently (Lenzenweger et al., 2001; Irwin, 2004; Dagnall et al., 2017). Although the IPO-RT possesses psychometric integrity, the degree to which the measure actually corresponds to real world situations has yet to be fully established. Additionally, because self-report responses index events retrospectively they are prone to forgetting and distortion (Afflerbach and Johnston, 1984). These concerns are not particular to the IPO-RT but apply to self-report measures generally. Until research validates the IPO-RT against objective measures of proneness to reality testing deficits, such as the Rorschach inkblot method, it is safer to conclude that the IPO-RT indexes subjective evaluation of the perceived likelihood of reality testing errors. The Rorschach inkblot method is a reliable index of perception of reality accuracy (Hilsenroth et al., 1998). This approach is consistent with the notion that psychopathological construct validation requires repeated assessment over time via a range of methods (Mason, 2015).

This is an important development because work in related psychopathological and cognitive domains has demonstrated that self-report measures designed to assess metacognitive processes often lack validity. For example, Searleman and Herrmann (1994) observed that self-report measures used to assess participant’s awareness of memory processes were reliable but failed to predict accurately memory abilities and use of metacognitive strategies (Searleman and Herrmann, 1994). This is because self-report measures, such as the IPO-RT, indirectly assess metacognitive processes. These are internal executive processes, which control, monitor and supervise cognitive processes (Sternberg, 1986). Metacognitive processes are vital to all stages of cognitive performance planning, monitoring, execution, and evaluation (Sternberg, 1986).

In the current paper, self-report measures assessed metacognitive strategies. A potential limitation of this approach arises from the fact that strategies employed by individuals may not be fully accessible to conscious awareness and therefore not reportable (e.g., Nisbet and Ross, 1980; Kentridge and Heywood, 2000; Koriat and Levy-Sadot, 2000; Dijksterhuis et al., 2006). Consequently, self-report methods only provide a partial and limited view of the potential operation of metacognitive processes. Ideally, the validity of self-report measures needs substantiating with other performance measures. This would be possible in situations where the operation of particular metacognitive strategies lead to definitive predictions on tests of cognitive performance (for example, the use of metacognitive monitoring on tests of memory, Dodson and Schacter, 2001). In situations similar to these, self-report measures of individuals are not required. Rather, inferences derive from actual performance measures. Experiments designed to exploit or promote the usage of particular metacognitive strategies would allow appropriate predictions regarding their deployment. In relation to the current work, and the use of self-report measures, a fuller and more complete understanding is achievable by assessing (i) the concordance between objective (performance) and self-report methods and (ii) the conditions under which these measures are congruent or diverge. Findings derived from self-report measures do not necessarily always deviate from performance measures; rather, their degree of congruence may be subject to a number of situational (experimental) constraints. In this context, important future research needs to examine the degree to which IPO-RT scores correspond to other performance-based reality testing measures.

An additional limitation relates to the use of CFA estimation method (i.e., ML). Beauducel and Herzberg (2006) compared ML estimation using Pearson correlations with weighted least squares mean and variance adjusted (WLSMV) estimation using polychoric correlations. WLSMV led to more accurate results for Likert scale-type data. Therefore, although bootstrapping helped to protect against standard error biases in this study, future research should consider using WLSMV estimation in factorial analyses of the IPO-RT.

Finally, further work needs to examine the temporal stability of the IPO-RT. Temporal stability is an important factor to consider when assessing the efficacy of self-report measures indexing personality disorders (Samuel et al., 2011). Evidence advises that there are age-related differences related to susceptibility to borderline personality disorder (BPD) (Zanarini et al., 2003). Specifically, clinical studies report relatively high remission rates (e.g., Zanarini et al., 2003). Extrapolating this finding to non-clinical samples there is reason to believe that both the tendencies to experience and report reality testing deficits is likely to change over time. Knowing the degree of alteration would indicate whether propensity to reality testing errors was dispositional or more transitory in nature. Dimensional scores showing consistency over time would indicate that reality testing, similarly to personality traits, represents a relatively stable individual difference in thinking/information processing style. Replicating the present results via test–retest reliability over lengthy periods would establish IPO-RT reliability and indicate whether the proposed bifactor solution was enduring. In this context, tests of scale stability and change are essential features of subsequent work.

## Author Contributions

AD and ND: theoretical focus, data analysis, and article development. AP: contributed to the writing process. KD: collected data and contributed to the writing process. RW: provided additional conceptual guidance and commentary.

## Conflict of Interest Statement

The authors declare that the research was conducted in the absence of any commercial or financial relationships that could be construed as a potential conflict of interest.
